# Challenges imposed by minor reference alleles on the identification and reporting of clinical variants from exome data

**DOI:** 10.1186/s12864-018-4433-3

**Published:** 2018-01-15

**Authors:** Mahmoud Koko, Mohammed O. E. Abdallah, Mutaz Amin, Muntaser Ibrahim

**Affiliations:** 1 Department of Molecular Biology, Institute of Endemic Diseases, University of Khartoum, P. O. Box 102, Army Road, 11111 Khartoum, Sudan; 2 Department of Neurology and Epileptology, Hertie Institute for Clinical Brain Research, Tübingen, Germany; 3 Department of Biochemistry, Faculty of Medicine, University of Khartoum, Khartoum, Sudan

**Keywords:** Minor reference alleles, Variant calling, Human exome, Next generation sequencing

## Abstract

**Background:**

The conventional variant calling of pathogenic alleles in exome and genome sequencing requires the presence of the non-pathogenic alleles as genome references. This hinders the correct identification of variants with minor and/or pathogenic reference alleles warranting additional approaches for variant calling.

**Results:**

More than 26,000 Exome Aggregation Consortium (ExAC) variants have a minor reference allele including variants with known ClinVar disease alleles. For instance, in a number of variants related to clotting disorders, the phenotype-associated allele is a human genome reference allele (rs6025, rs6003, rs1799983, and rs2227564 using the assembly hg19). We highlighted how the current variant calling standards miss homozygous reference disease variants in these sites and provided a bioinformatic panel that can be used to screen these variants using commonly available variant callers. We present exome sequencing results from an individual with venous thrombosis to emphasize how pathogenic alleles in clinically relevant variants escape variant calling while non-pathogenic alleles are detected.

**Conclusions:**

This article highlights the importance of specialized variant calling strategies in clinical variants with minor reference alleles especially in the context of personal genomes and exomes. We provide here a simple strategy to screen potential disease-causing variants when present in homozygous reference state.

**Electronic supplementary material:**

The online version of this article (10.1186/s12864-018-4433-3) contains supplementary material, which is available to authorized users.

## Background

With the current genomic revolution, an extra burden is placed on researchers and clinicians to familiarize themselves with the common caveats faced in the field. One perplexing issue is how to choose the best reference not only for the species but for each variation site as well. Although the term “variations” implies differences between individuals and populations, the choice of a single reference is a practicality imposed by the need for a common ground for the analysis. The alignment of sequences to this reference is tolerant to variations. However, the basic concept of variant calling is to contrast the sequence reads against a reference to detect mismatches as variants. Choosing the best possible combination of reference alleles to each and all variation sites in the human genome is thus critical to the frictionless calling, annotation and prioritization of variations. The fact remains, however, that many variation sites in the human genome harbor an allele denoted as a reference – with a frequency lesser than the other alternate allele(s) in the same position [1]. Following the worldwide efforts to genotype and sequence a more representative number of human genomes and exomes e.g. 1000 Genomes Project [2] and Exome Aggregation Consortium (ExAC) project [3], it was clear that many of the reference alleles in the Human Genome Project were rare or otherwise population specific. These sites, which we describe hereafter as “minor reference alleles”, are challenging during variant calling.

The accepted standards for variant calling, e.g. the Genome Analysis Tool Kit best practices [4–6], consider the choice of an appropriate reference a part of the research question and study design. Variant calling usually treats homozygous reference alleles as “no change”. Generally, no distinction is made between variation sites and the rest of the human genome where no variation is observed. On the other hand, the guidelines for interpretation and reporting of variants tackle only the proper annotation of already detected variants. The Human Genome Variation Society (HGVS) sequence nomenclature [7] is considered by many researchers the primary consensus to report genetics variations. The HGVS nomenclature standard states that the recommended reference sequence is “a genomic reference sequence based on a recent genome build”. The reference alleles are to be reported as “no change” using “=” sign (e.g. c.1G = means reporting the reference allele at the first cDNA position). However, these guidelines are based on the assumption of using an appropriate reference sequence that represents the “normal” state. The American College of Medical Genetics (ACMG) guidelines for the interpretation of sequence variants [8] do not explicitly require the investigators to evaluate or report minor/rare homozygous reference variations. Up to date, there are no clear recommendations for the identification of homozygous reference variants in exome and genome experiments.

Magi et al. [1] studied the count and annotation of “rare” reference alleles in 1000 Genomes data and developed a specialized variant caller (RAREVATOR). Dewey et al. [9] used a modified version of the human genome as a “major allele” population specific reference. These approaches have some limitations as we discuss later. We studied the latest release of ExAC data as a more extensive source for coding variants [3] to find out the extent of minor reference alleles in the human genome and their implication on variant calling. Here, a simple flexible strategy is proposed to interrogate these variations based on a bioinformatic panel of minor reference allele sites, which we tested using the publicly available Genome In A Bottle [10] (GIAB) datasets. Additionally, exome sequencing data from an individual with a thrombotic tendency is presented to discuss how the presence of minor reference alleles in the human genome affected the identification and reporting of relevant ClinVar alleles [11] (ClinVar is a widely used public archive of the clinical relevance of human variations that aggregates information about variations observed in individuals with or without a phenotype).

## Methods

### Minor Reference Alleles

Minor reference allele (MRA) variants were defined as variants where one of the alternate alleles is commoner than the reference (reference allele frequency less than 0.5 after multiple alleles splitting). A pair-wise comparison of frequencies between reference alleles and all observed alternate alleles from the ExAC [3] release 0.3.1 VCF file was performed. Variants with multiple alleles were split using vt tool [12] v0.5772 with sub-setting allele specific frequencies. All variants were then filtered based on an allele frequency cut-off = 0.5. The resulting coordinates and alleles information were formatted as both VCF and BED. bcftools [13] and bedtools [14] were used for the handling of VCF and BED files and calculating overlaps. The genomic coordinates were lifted-over to hg38 using NCBI remapping service [15] to obtain the human genome assembly 38 equivalent coordinates, followed by removing sites with reference update and decoy remaps. Those ExAC minor reference variants were used as a bioinformatic panel for direct genotyping of minor reference sites (considered non-variation sites by the variant callers) followed by filtering for homozygous reference alleles (see Additional files 1 and 2). The combination of allele genotyping through targeted calling and filtering for homozygous reference alleles allows to screen homozygous reference variants using any variant caller that supports targeted calling. This approach can be implemented in principle by targeted calling in specified variation regions which can be specified based on global or population-based ExAC allele frequencies.

### Testing using the Genome Analysis Toolkit against GIAB data

Two publicly available reference exome sequencing binary alignment (bam) files from the sample NA12878 (NIST v3.3.2) that are characterized for homozygous reference regions were used for targeted calling. The exomes of NA12878 are reference materials available from the GAIB consortium. The two exomes were sequenced from one DNA sample enriched using nextrarapid library on two lanes on Illumina HiSeq2500 platform (Illumina, San Diego, CA, US), aligned using novoalign (Novocraft Technologies, Malaysia), and preprocessed by GIAB according to widely accepted Genome Analysis Toolkit (GATK v2.6) best practices. The VCF file of ExAC minor reference alleles described above was subset for the nextrarapid enrichment regions and was used as input to GATK v3.4.46 Haplotypecaller which uses a haplotype-based variant calling strategy to perform local re-assembly of reads around variation sites and subsequently identify short variants (single nucleotide variants and short deletion/insertions) in the form of genotype blocks. GATK Haplotypecaller tool was used in two modes: “discovery” mode to obtain reference genotype qualities and “genotype given alleles” mode to limit genotyping to the panel alleles. Variants were then filtered for homozygous reference alleles with a depth (DP) > 20 and reference genotype quality (RGQ) > 30 (equals a wrong genotype call probability of 0.001). As site qualities (QUAL field in VCF) are set to zero or negative at reference sites by most variant callers, we evaluated the RGQ (reference genotype qualities) and genotype qualities (GT) as the main quality metric for quality control that will be correctly calculated. The complete list of variants is supplied as Additional file 3.

### Testing in a thrombophilia patient

Whole exome sequencing data from a Sudanese male (see data availability) with a thrombotic tendency was analyzed using the same approach detailed above. He had a medical history of pulmonary embolism. His family history was significant for atopy and ischemic heart disease in first degree relatives. His previous investigations included genotyping of three thrombophilia high risk variants. Genomic DNA was extracted from a peripheral blood sample and assessed for quality using agarose gel electrophoresis and spectrophotometry. Exome sequencing was performed at Beijing Genomics Institute (BGI, Hong Kong). Agilent SureSelect Human All Exon enrichment kits (Agilent, CA, US) were used with a total target size of 50.4 Mbases. Paired-end sequencing was performed on two lanes on Illumina HiSeq2500 platform. A total of 14,207,118 filtered read pairs were available with a median length of 100 bp and non-duplicated reads percentage of 96.76%. Alignment was performed on a public Galaxy platform [16] (open web-based platform for biomedical informatics). The sequence read (fastq) files were aligned to the human genome assemblies hg19 and hg38 (with decoy contigs) using Galaxy bwa v0.7.12.1 (burrows-wheeler aligner is a popular open-access aligner [17] for short and medium-length reads). Around 94% of reads were mapped uniquely to the human genome at a quality ≥20 with a mean coverage of 33× (with 71% of mapped bases inside or near target regions). The bam files were sorted with removal of duplicates using Galaxy samtools [13] v0.2. GATK v3.4.46 was utilized for realignment around indels and base quality score recalibration, followed by standard variant calling using Haplotypecaller. GATK GenotypeGVCFs tool was used to genotype the SNVs and Indels in these genotype blocks. The resulting VCF files were then split for multiple alleles and normalized using vt v0.5772 then annotated using Ensembl Variant Effect Predictor [18] (VEP) v88, a tool for variant annotation that provides a wide range of annotations including affected genes, frequency of variants, consequences on the transcripts and proteins, among other annotations. NCBI ClinVar release 201,607 annotations were obtained. For homozygous minor reference alleles identification, variant calling with the minor reference allele panel was performed as described above for NA12878. To prioritize clinically relevant candidates, a list of genes causing bleeding diathesis (including genes with a known thrombosis phenotype) was compiled from multiple reviews and reports [19–23] collected through NCBI PubMed [24] and OMIM [25] search. The genes were: *ACE, ANGPT1, ANGPTL4, APOH, AVPR2, B4GALT1, CCR2, CD40LG, CPB2, F10, F11, F12, F13A1, F13B, F2, F3, F5, F7, F8, F9, FGA, FGB, FGG, GGCX, GP1BA, GP6, HABP2, HGF, HRG, ITGA2B, JAK2, LPA, MMADHC, MST1, MTHFR, MTR, MTRR, NOS3, P2RY12, PLAT, PLAU, PLG, PROC, PROS1, RAPGEF1, SERPINC1, SERPIND1, SERPINE1, TFPI, THBD, VWF*. The coordinates of these genes were obtained using Ensembl BioMart [26]. All variants in these genes (detected with standard calling or minor reference allele calling) were evaluated for candidates that might increase the risk of thrombosis.

## Results

We found that the latest ExAC release contained 26,537 variants with a reference allele frequency less than 0.5. Around 1% of these variants (2763 variants) were rare variants (AF < 0.01). Out of this pool, a group of variants had a ClinVar significance of (likely)pathogenic, risk factor, association, or drug response allele (Table 1). Lift-over to the new assembly hg38 indicated that 1214 known variants had an updated reference allele (Additional files 1 and 2).

**Table 1:** Some ClinVar variants with minor reference alleles. Allele frequencies are reported from the Exome Aggregation Consortium (ExAC)

rsID	REF		ExAC Minor Allele		Amino-acid change	Conservation		ClinVar phenotype or Disease		
	hg19	hg38	Allele	MAF	Ref/Alt	Rhesus	Mouse	Amino-acid	phenotype	risk
rs1169305	A	A	A	0.004	S/G	G	N	S	Maturity onset diabetes	pathogenic
rs4784677	C	C	C	0.006	S/N	N	N	S	Bardet-biedl syndrome	pathogenic
rs497116	**C**	**T**	**T**	0.014	R/Q	Q	Q	R	Sepsis	risk factor
rs6025	**T**	**C**	**T**	0.02	Q/R	R	Q	Q	Factor V Leiden	pathogenic
rs283413	**A**	**C**	**A**	0.02	T/P	P	P	T	Parkinson disease	risk factor
rs820878	T	T	T	0.03	L/S	S	S	L	Sandhoff disease	pathogenic
rs2476601	A	A	A	0.07	W/R	R	R	W	(multiple autoimmune diseases)	risk factor
rs450046	C	C	C	0.08	R/Q	–	Q	R	Proline Dehydrogenase deficiency	pathogenic
rs12021720	T	T	T	0.09	S/G	G	G	S	Maple syrup urine disease	pathogenic
rs6003	C	C	C	0.13	R/H	H	H	R	Factor XII deficiency	pathogenic
rs1154510	T	T	T	0.15	T/A	A	A	T	Hawkinsinuria	pathogenic
rs7076156	A	A	A	0.21	A/T	G	–	A	Nephrolithiasis	risk factor
rs1801265	**G**	**A**	**G**	0.23	R/C	R	R	R	Dihydropyrimidine dehydrogenase deficiency	pathogenic
rs1799983	T	T	T	0.25	D/E	E	E	D	Ischemic heart disease	risk factor
rs2227564	T	T	T	0.25	L/P	Q	Q	L	Alzheimer disease	risk factor
rs1061170	C	C	C	0.33	H/Y	N	W	H	Basal lamina drusen	pathogenic
rs1341667	T	T	T	0.38	Y/H	Y	Y	H	Pre-eclampsia	risk factor
rs2073711	A	A	A	0.43	I/T	I	I	T	Lumbar disc disease	risk factor
rs237025	G	G	G	0.44	V/M	M	–	V	Diabetes mellitus	risk factor
rs3733402	G	G	G	0.46	S/N	N	N	S	Prekallikrein deficiency	pathogenic

Alleles that changed between assemblies are in bold font

Reference alleles with low allele frequencies are sites of mismatch for the majority of human populations, appearing frequently as homozygous alternate (or heterozygous) variants with high alternate allele frequency. When samples are studied individually especially in the context of clinical genomes and exomes, an individual with a common genotype will have homozygous alternative variants which is usually non-pathogenic. In other words, because the alternative alleles are commoner than the reference ones, all whole exome (and genome) variant calling results usually show a number of variants with high alternate allele frequencies (the alleles that are seen in the sequenced samples are major alleles) but low minor allele frequencies (the reference alleles are minor or even rare). These sites are usually filtered upon prioritization in disease studies. Using standard calling in the exome set from the thrombophilia patient, multiple clinically relevant variants in homozygous alternative (rs6050, rs2066865, rs6025, rs2815822, rs2227564) and heterozygous states (rs6003, rs17549873, rs1800595, rs1799810, rs1063856) were seen (Table 2). Fibrinogen alpha and gamma variants (rs6050 and rs2066865, inherited mostly together in a linkage disequilibrium block) are known to increase the thrombotic risk and likely explain the thrombotic state in this patient. However, the other variants (rs6025, rs2815822, rs2227564) were “calling artifacts” caused by their minor reference alleles.

**Table 2:** Variants with disease-associated reference alleles in thrombophilia genes

Gene	Variant	Minor Allele Frequency			Reference alleles		Disease allele	ClinVar variants
		1000G	ExAC	Allele	hg19	hg38		
F5	rs6025	0.0060	0.0215	T	T	C	A	c.1601G > A, c.1601G=
F13B	rs6003	0.2382	0.1280	C	C	C	G	c.344G > A
PLAU	rs2227564	0.2246	0.2454	T	T	T	T	c.371C > T
NOS3	rs1799983	0.1763	0.2470	T	T	T	T	c.894 T > G

The pathogenic rs6025 Leiden variant [27] is c.1601G > A (p.Arg534Gln). The minor allele A is the reference allele in hg19. In this thrombophilia exome, we encountered a homozygous alternative (G/G) variant call in rs6025 (non-pathogenic allele) when exome sequencing reads were aligned to hg19. No call was seen upon alignment to hg38, where the genotype was confirmed also to be G/G upon reads visualization. Clinical variations in factor 13 subunits predispose to bleeding diathesis and possibly thrombosis [28–31]. Variations in the *F13A1* gene that gives the active subunit of Factor XIII protein are linked to bleeding tendencies (Factor XIIIA deficiency), protection against venous thrombosis, and protection against myocardial infarction [28]. In ClinVar, rs2815822 is reported as (c.-19 + 12A=) and linked to deficiency of the A subunit of Factor XIII. The reference A is the phenotype-associated allele. The phenotype of this individual (thrombotic tendency) was not in conformity with factor 13 A subunit deficiency (bleeding tendency, protection against thrombosis). In line with this observation, he had a homozygous non-pathogenic G/G genotype. As well, a recent pathogenicity review in ClinVar classified the variant as benign. Additionally, variants in Urokinase-type Plasminogen Activator *(PLAU)* gene have been reported to associate with collateral circulation in patients with coronary artery disease with conflicting evidence [32, 33] although it has not been directly associated with venous thrombotic tendencies (different phenotype in ClinVar). The reference T allele of the variant rs2227564 was found to be more common in patients with poor collateral circulation. This variant was detected in homozygous non-pathogenic C/C genotype in this individual.

The mirror image of this scenario is an individual with a homozygous reference variant in one of these sites. Such a reference allele that might have a clinical relevance will be missed during variant calling in affected individuals harboring the less-frequent (but reference) genotype in homozygous state as the variant caller will not observe any difference to the reference. Additionally, the majority of reports using exome sequencing do not evaluate homozygous reference alleles. Keeping in mind that most studies look for alleles with very low frequencies in the human genome [34–36], these sites will be missed during conventional variant calling. In rare disorders, such sites are likely benign because they are seen in normal reference individuals. They become increasingly relevant for all diseases where common variants (allele frequencies >1%) can cause or modulate the risk of disease.

Using targeted calling for minor reference alleles, 1043 homozygous MRA variants were genotyped in the GIAB sample NA12878. In total, 692 variants overlapped regions where GIAB offers high confidence calls and were used to assess the performance of this approach. The concordance rate with the high confidence calls was 91.62% while the variant calls were different for 58 out of 692 variants (8.38%). With a depth threshold of 20 and genotype quality threshold of 30, a total of 627 MRA variants overlapped high confidence calling areas while the number of false homozygous calls dropped down to 40 (6.38%). All of these “false” calls were sites of reference allele mismatch, position mismatch, or low complexity sequences (e.g. repeats). It is worth mentioning that the confidence calls are made from an array of sequencing reads from different platforms and at higher total depth than the single set we used for testing. The complete lists of these variants are provided in Additional file 3.

In the thrombophilia exome set, the use of a minor reference panel successfully detected a ClinVar variant rs1799983. The patient had a T/T homozygous genotype confirmed by visualization of reads alignment. The variant rs1799983 codes a missense Glu298Asp change in exon 7 of the Nitric Oxide Synthase 3 (*NOS3*) gene which is linked to coronary artery spasm, ischemic heart disease, ischemic stroke and resistant hypertension [37, 38]. It is related to the clinical history of this individual as it was reported to predispose to venous thrombosis [39–43] and relates to his family history of ischemic heart disease.

## Discussion

The advantages of performing panel and whole exome sequencing in comparison to “risk alleles” genotyping has been reviewed extensively. In this Sudanese individual, such small-scale genotyping of three common risk variants (rs7080536, rs1799963, rs6050) concluded that his condition is linked to a homozygous *FGA* 6534G allele (rs6050). The exome sequencing provided more detailed account of his risk profile by detecting another homozygous *FGG* variant (rs2066865) with a similar or higher risk and multiple heterozygous variants with variable risk odd-ratios. However, these advantages are accompanied by other challenges. A number of thrombophilia variants have minor reference alleles. The classical example is Factor V Leiden variant rs6025. Other variants with pathogenic reference alleles include: rs6003, rs2227564 and rs1799983.

Reference allele updates between assemblies have resolved the issue of pathogenic minor reference alleles for some but not all human variations. The reference allele for Factor V Leiden rs6025 variant is updated in the current hg38 genome build to be the common allele G and thus an affected patient with A/A genotype will be correctly identified. For other variants with no reference update, both assemblies still face the dilemma of missing homozygous reference disease-associated genotypes. Moreover, technical barriers hinder the quick transition between assemblies. Although the current human genome assembly 38 is taking over as a default, many researchers still favor the use of the previous assembly. It is clear that this transition will take a while, starting from the targets of enrichment kits that are commonly provided in hg19 coordinates and going all the way to popular open-access databases (e.g. ExAC) that provide to date hg19-aligned genomic data.

It is noteworthy that the allele frequency of most of these variants is relatively high. This high frequency is possibly related to their variable penetrance and effect size. The gap in variant calling probably affects common alleles more than rare alleles, making it more likely to miss a sizable number of patients if assessed individually. Among 3556 disease-susceptibility alleles reported by Chen et al. [44] as human genome reference alleles, only 15 were rare. As well, in more than 26,000 ExAC variants with minor reference alleles, only 1% are rare. This is rather expected as the rare and ultra-rare variants in the human population tend to associate with severe phenotypes (the reference genome samples were taken from apparently healthy individuals). Further, it explains why the exclusion of homozygous references in exome (or genome) studies in patients with rare diseases is understandable under a rare-variant rare-disease model. On the contrary, when common disorders are investigated, researchers should be more careful about discarding homozygous references. Missing homozygous pathogenic reference variants will result in inaccurate risk determination and diagnosis, especially when another variant is seen in standard variant calling, leading to premature conclusions.

As mentioned, variant callers will report only variations to the reference unless specifically requested to do otherwise. When multiple patient and control samples are evaluated together in joint calling or otherwise reference-free variant calling [45], this limitation in calling reference variants is overcome by making genotype comparisons between the samples themselves (in the presence or absence of a reference, respectively). Yet, it is not uncommon to evaluate single samples. In this case, directly genotyping reference alleles as described here, using specialized variant callers [1], or modified reference genomes [9] (e.g. population specific genomes or major-allele genomes where the reference alleles have been swapped at minor reference allele positions) provide work-arounds. A specialized variant caller, RAREVATOR [1], was designed to evaluate only rare variation positions and would not evaluate common polymorphisms. Additionally, it is based on the Unifiedgenotyper algorithm of GATK which has been recently replaced by the haplotype-based algorithm of Haplotypecaller. The use of modified genomes – although very appealing – has downsides: the annotation and sharing of variants will be difficult as most if not all of the available genomic databases require reference consistency to provide accurate annotations. An extra step of normalization (checking of reference allele consistency and coordinates) will be needed to join or compare VCF datasets called against different references. Targeted calling is a very flexible strategy that can utilize panels customized to the research question in hand (e.g. based on a panel of genes or population based frequencies). The minor reference alleles dilemma is compounded by the multiplicity of populations “private” alleles. The panel approach comes handy as the variants can be defined using population-based frequencies that can be easily updated with the expanded wealth of genomic databases. As quality measures, read depth (the number of times a site is overlapped by non-duplicate reads) and genotype qualities (probability of a correct genotype call) can be used to estimate the confidence of the resulting homozygous reference calls. To prioritize the results, variants in candidate genes or genes that have biological relevance to the phenotype can be investigated. Downstream filtering may make use of amino-acid conservation information. It has been shown than minor non-ancestral alleles tend to be functional [46]. The non-pathogenic alternative alleles tend to give conserved amino-acids while the reference (disease-associated) alleles tend to represent amino-acids that are different from other closely related species.

Finding disease-associated genetic variants is arguably the largest utility of the human genome so far. Tuning the reference genome to improve its clinical utility should have a considerable weight in defining reference alleles. By incorporating phenotypic annotations, alleles with a large pathogenic effect size (e.g. associated with proven increase in disease relative risk) should always be described as alternative alleles. Some complex areas in the genome would remain difficult to take as a reference for all the human population. Finding a convention to define the best reference allele will help immensely to improve the “human genome” in its new updates and to provide a correction of these minor reference alleles. An objective or at least consensus definition regarding what should be considered a reference allele is matter of ongoing debate. Evidence from multiple layers should be involved. For instance, disease alleles were found to be mostly minor and derived [47]. Nonetheless, allele frequencies have to be taken within the context of population history and ancestry [48]. A major ancestral allele (or that have a higher frequency in ancestral populations) would make a better choice as a reference from an evolutionary perspective.

However, there are multiple examples of mutations that occurred in Africa with frequencies that went up through directional selection among non-Africans while remaining low in Africans. Hence establishing ancestral genomes as a baseline is considered important. On the other hand, the presence of a single human genome is important for a unified “technical” description and reporting of variations. The provision of population-based reference contigs for such complex areas is one way to help us enjoy the benefits of a single genome while accommodating a broader range of variation. The human genome hg38 release has witnessed a large increase in the number of alternative and decoy contigs (e.g. in Major Histocompatibility Complex gene clusters) which improves the alignment quality and subsequently the calling quality. Like alternate-contigs-aware aligners, variant callers with support for alternate contigs are an appealing possibility.

## Conclusions

The identification and reporting of homozygous reference variants can be of clinical value. In these sites, homozygous alternate variants tend to represent the non-pathogenic allele. On the other hand, the homozygous reference alleles, which are usually overlooked, may bear direct clinical implications. These variants should be evaluated by modified calling strategies especially in the setting of personal exomes and genomes. As a long-term solution, a consequences definition of the “reference allele” state in the human genome is needed to provide global yet comprehensive future genome assemblies.

## Additional files

Additional file 1: ExAC_MRA.xls. Minor reference alleles in ExAC database. (XLS 5024 kb)

Additional file 2: ExAC_MRA_vcf.xls. ExAC minor reference alleles aligned to hg19 and hg38 in VCF formatting. The tabular data can be exported as tab separated files with vcf extension for use with standard VCF file viewers. (XLS 7347 kb)

Additional file 3: NA12878_MRA.xls. Homozygous minor reference alleles in GIAB NA12878 reference sample. (XLS 395 kb)

## Abbreviations

ACMG: American College of Medical Genetics
BAM: Binary Alignment Map format
BED: Browser Extensible Data format
ExAC: Exome Aggregation Consortium
GATK: Genome Analysis Tool Kit
GIAB: Genome In A Bottle consortium
HGVS: Human Genome Variation Society
MRA: Minor Reference Alleles
NCBI: National Center for Biotechnology Information
OMIM: Online Mendelian Inheritance in Man database
VCF: Variant Calls Format
